# Distinct landscapes of fibroblast subtypes in arteries of patients with giant cell arteritis

**DOI:** 10.1093/rheumatology/keaf143

**Published:** 2025-03-14

**Authors:** Shuang Xu, William F Jiemy, Yannick van Sleen, Johanna Westra, Jacoba C Graver, Kornelis S M van der Geest, Peter Heeringa, Annemieke M H Boots, Elisabeth Brouwer, Maria Sandovici

**Affiliations:** Department of Rheumatology and Clinical Immunology, University Medical Center Groningen, University of Groningen, Groningen, The Netherlands; Department of Rheumatology and Clinical Immunology, University Medical Center Groningen, University of Groningen, Groningen, The Netherlands; Department of Rheumatology and Clinical Immunology, University Medical Center Groningen, University of Groningen, Groningen, The Netherlands; Department of Rheumatology and Clinical Immunology, University Medical Center Groningen, University of Groningen, Groningen, The Netherlands; Department of Rheumatology and Clinical Immunology, University Medical Center Groningen, University of Groningen, Groningen, The Netherlands; Department of Rheumatology and Clinical Immunology, University Medical Center Groningen, University of Groningen, Groningen, The Netherlands; Department of Pathology and Medical Biology, University Medical Center Groningen, University of Groningen, Groningen, The Netherlands; Department of Rheumatology and Clinical Immunology, University Medical Center Groningen, University of Groningen, Groningen, The Netherlands; Department of Rheumatology and Clinical Immunology, University Medical Center Groningen, University of Groningen, Groningen, The Netherlands; Department of Rheumatology and Clinical Immunology, University Medical Center Groningen, University of Groningen, Groningen, The Netherlands

**Keywords:** giant cell arteritis, fibroblast subtypes, vascular remodelling

## Abstract

**Objective:**

Giant cell arteritis (GCA) is a systemic vasculitis of large- and medium-sized arteries characterized by granulomatous inflammation and vascular remodelling. Although fibroblasts are the predominant cell type in the adventitia, their role in GCA pathogenesis is largely unknown. This study aimed to investigate the distribution of fibroblast subtypes in relation to vascular remodelling in GCA.

**Methods:**

Temporal artery biopsies (TAB) from patients with GCA (*n* = 9) and controls (*n* = 15) and aorta tissues from GCA (*n* = 9)- and atherosclerosis (*n* = 11)-related aneurysms were examined. Immunohistochemical and immunofluorescence staining for fibroblast subtype markers (CD90, platelet-derived growth factor receptor α [PDGFRA], fibroblast activation protein [FAP], podoplanin [PDPN], CD248, α-smooth muscle actin [α-SMA]), cellular proliferation (Ki67) and remodelling-related growth factors (TGF-β, fibroblast growth factor 21 [FGF21], platelet-derived growth factor B [PDGFB]) were performed to evaluate the distribution of fibroblast subtypes in relation to active remodelling pathways. To evaluate the role of FAP in TGF-β-induced fibroblast proliferation, human aortic adventitial fibroblasts (HAoAF) were stimulated *in vitro* with TGF-β and transfected with small interfering RNA targeting FAP.

**Results:**

In GCA-TAB, CD90^+^FAP^+^ activated fibroblasts and CD90^+^PDPN^+^ immunofibroblasts were predominantly located in the adventitia. CD90^+^α-SMA^+^ myofibroblasts were observed mainly in the intima, and CD90^+^CD248^+^ fibroblasts in the adventitia–media border and intima. High FGF21 and PDGFB expression in the intima was associated with intimal hyperplasia in GCA-positive TAB. GCA-affected aortas showed a different landscape of fibroblast subtypes: CD90^+^FAP^+^ activated fibroblasts, CD90^+^PDPN^+^ immunofibroblasts and CD90^+^CD248^+^ fibroblasts accumulated especially in structurally disrupted media. Approximately 80% of proliferative fibroblasts in TAB and aorta were FAP positive. FAP knockdown suppressed TGFβ-induced proliferation of HAoAF *in vitro*.

**Conclusion:**

This study documents a distinct spatial distribution pattern of fibroblast subtypes in GCA-affected arteries. The data suggest different roles for fibroblasts in remodelling of specific arterial vascular beds in GCA.

## Graphical abstract

**Figure:**
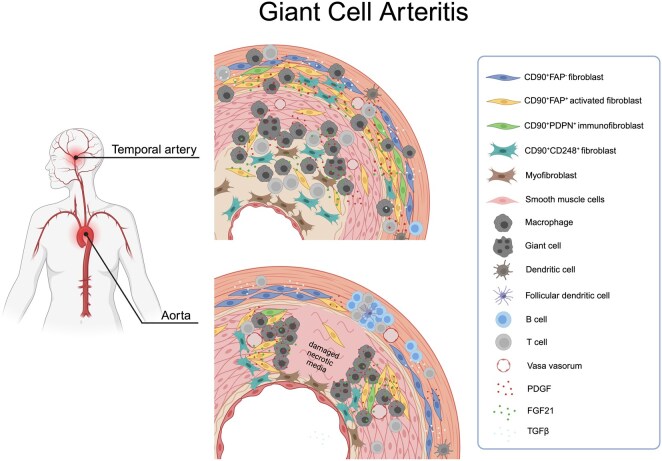
Fibroblast subtypes and their distinct distribution pattern in temporal artery and aorta of patients with giant cell arteritis (made with BioRender.com)

Rheumatology key messages

- Temporal artery and aorta present distinct distribution patterns of fibroblast subsets in GCA.
- Cell proliferation is more important than collagen production for vascular remodelling in GCA.
- Fibroblasts proliferate at the sites of vascular remodelling in GCA, a process seemingly related to expression of fibroblast activation protein.

## Introduction

Giant cell arteritis (GCA), the most frequent form of systemic vasculitis in people over 50 years of age [1], is characterized by granulomatous inflammation and vascular remodelling of medium- and large-sized arteries, including the temporal artery (cranial GCA) and aorta (large vessel GCA). The pathogenesis of GCA is postulated to start in the adventitia, initiated by vascular resident dendritic cell (DC) activation, followed by CD4^+^ T cell recruitment, activation and polarization [2, 3] mainly towards T helper (Th)1 and Th17 cells. The Th1/Th17 axes further mediate monocyte recruitment and macrophage activation [4] by chemokine and cytokine production. Inflammation triggers vascular remodelling through release of cytokines, growth factors and matrix metalloproteinases (MMPs), eventually contributing to aneurysm formation in the aorta and intimal hyperplasia and stenosis of the vascular lumen in its branches [5]. Important known cellular players in the remodelling process in GCA are myofibroblasts/vascular smooth muscle cells (VSMC) and macrophages, whereas the role of adventitial fibroblasts remains largely unexplored. Important molecular signals in the remodelling process include transforming growth factor β (TGF-β), platelet-derived growth factor B (PDGFB) and fibroblast growth factor 21 (FGF21).

Fibroblasts are important players in tissue healing and remodelling after mechanical- and inflammation-induced injury. They are traditionally recognized for their role in synthesizing and remodelling the extracellular matrix (ECM), thereby maintaining tissue architecture [6]. With the advances in molecular phenotyping over the last decade, the heterogeneity of fibroblasts has been recognized. Several markers, such as CD90, platelet-derived growth factor receptor α (PDGFRA), and collagen I α chain (COL1A), are considered pan-fibroblast markers [7, 8]. α-Smooth muscle actin (α-SMA) is a marker of myofibroblasts, rendering cells capable of migration [9]. Fibroblast activation protein α (FAP) characterizes activated fibroblasts and is important in regulating proliferation, migration and angiogenesis [10]. CD248 is a marker of pro-fibrotic fibroblasts; CD90^+^CD248^+^ fibroblasts may indicate an intermediate status between quiescent fibroblasts and myofibroblasts [11, 12]. Expression of podoplanin (PDPN) by fibroblasts has been linked to an immune-active subtype of fibroblasts, the so-called immunofibroblasts [13]. CD90^+^PDPN^+^ immunofibroblasts may be involved in immune regulation in GCA [14, 15] (Table 1). Besides their role in synthesizing and renewing the ECM, fibroblasts can also influence the surrounding cellular and molecular environment by producing cytokines, chemokines and growth factors, as well as by engaging in cell–cell interactions [6, 16, 17].

**Table 1.** Distinct fibroblast markers

Markers	Fibroblast type	Functions
CD90, PDGFRA, COL1A1, vimentin	Pan-fibroblast	Tissue structure and maintenance of ECM homeostasis
FAP	Activated fibroblast	Cytokines/growth factors/ECM/MMPs production
PDPN	Immunofibroblast	Immunoregulation, cytokines/growth factors/ECM production
CD248	Pro-fibrotic	Pro-fibrosis
α-SMA	Myofibroblast	Tissue remodelling, cytokines/growth factors/ECM production
Desmin	Non-fibroblasts	—

α-SMA: α-smooth muscle actin; COL1A1: collagen I α chain; ECM: extracellular matrix; FAP: fibroblast activation protein α; PDGFRA: platelet derived growth factor receptor α; PDPN: podoplanin.

Although fibroblasts are dominant in the adventitia of medium- and large-sized arteries, the site where the vasculitic process is thought to initiate, their role in the pathogenesis of GCA is largely unknown [18]. Parreau *et al.* documented a significant increase in expression of fibroblast (CD90) and myofibroblast (α-SMA) markers in the intima and adventitia of GCA-affected temporal artery biopsies (TAB) compared with control TABs [19]. We recently reported altered circulating and tissue FAP expression levels in patients with GCA [20]. Furthermore, CD90^+^FAP^+^ fibroblasts in GCA-affected blood vessels expressed IL-6 and MMP-9, suggesting their involvement in both inflammatory and remodelling processes in GCA. As such, persistent fibroblast activation may underpin disease chronicity in GCA. In this study, we aimed to characterize the distribution of various fibroblast subtypes and their possible links to vascular remodelling in GCA. Our results collectively document distinct distribution patterns of fibroblast subtypes in GCA-affected temporal artery and aorta.

## Methods

### Study population

To study fibroblast distribution in tissues, inflamed TAB samples of histologically proven GCA patients (*n* = 9), non-inflamed TAB samples (*n* = 15; obtained from patients with isolated polymyalgia rheumatica [PMR; *n* = 5], non-PMR/non-GCA patients [*n* = 7], and negative artery biopsies from GCA-positive patients [*n* = 3]), and aortic tissues from patients with GCA-related aneurysm (*n* = 9) and atherosclerosis-related aneurysm (*n* = 11) who underwent aortic aneurysm surgery were studied (Table 2). All procedures complied with the Declaration of Helsinki. For use of the aorta tissue, consent from the Internal Review Board and written patient consent are not required under the Dutch law for human medical research (WMO) since this is a retrospective, non-interventional study. Patients were informed that their medical data or tissues could be used for research purposes in accordance with privacy rules. (UMCG Research Register Number: 201800370.)

**Table 2.** Characteristics of patients and control groups included in tissue analysis

Characteristic	TAB-GCA	TAB control	Aorta-GCA	Aorta-AS
No. of patients	9	15	9	11
Age, median (range), years	72 (59–81)	71 (51–86)	67 (55–79)	65 (59–78)
Gender, female (%)	88.9%	66.7%	66.7%	63.6%
No. of subjects on treatment with glucocorticoid/immunosupression	2	2	0	0
ESR, median (range), mm/h	46 (9-104)	52 (14-74)	24 (4-37)	17 (1-40)
CRP, median (range), mg/l	22 (4-158)	29 (4-87)	5 (1.4-52)	9 (3-32)

For two GCA-positive TAB, one patient was treated with prednisolone for 12 days until TAB; another patient was treated with prednisolone because of PMR (time period unknown) and stopped 1 week before TAB; For two GCA-negative TAB, one patient was treated for 5 years until TAB; another patient was treated with glucocorticoids for 5 months and stopped 5 days before TAB. AS: atherosclerosis; GCA: giant cell arteritis; TAB: temporal artery biopsy; PMR: polymyalgia rheumatica.

### Immunohistochemistry staining, immunofluorescence staining and Masson’s trichrome staining

Staining was performed to study various fibroblast phenotypes (CD90, FAP, PDPN, CD248, α-SMA, PDGFRA), remodelling (TGF-β, FGF21, PDGFB, collagen) and proliferation (Ki67) in GCA-affected vessels. Detailed methods can be found in Supplementary Data S1, available at *Rheumatology* online. For the list of antibodies and related protocols, see Supplementary Tables S1 and S2, available at *Rheumatology* online.

### Cell culture and transfection

Human aortic adventitia fibroblasts (HAoAF, C-12380) were obtained from PromoCell, Heidelberg, Germany. HAoAF, originated from the thoracic aorta of a 50-year-old Caucasian male donor. Cells were routinely cultured in low glucose Dulbecco’s modified Eagle’s medium supplemented with 10% fetal bovine serum, 50 μg/ml gentamycin, 10 μg/ml Insulin-Transferrin-Selenium-Sodium Pyruvate (ITS-A), 1 ng/ml basic fibroblast growth factor and 500 μM vitamin C and were grown at 37°C in a 5% CO_2_ humidified chamber. Cells were seeded in six-well plates (with coverglass)/12-well plates at a density of 25 000 cells/cm^2^ and reached 50% confluency after 24 h. Cells were then transfected with FAP small interfering RNA (siRNA; assay ID: s5023, Silencer Select, Thermo Fisher Scientific, Waltham, MA, USA) and non-binding control siRNA (catalogue no. 4390843, Silencer Select; Thermo Fisher Scientific) using Lipofectamine 2000 (Thermo Fisher Scientific) in accordance with the manufacturer’s instructions. Six hours after transfection, culture medium was replaced and TGF-β1 (Thermo Fisher Scientific, 100–21) was added. Twenty-four hours later, the inhibitory efficiency of these siRNAs was verified by qPCR. Seventy-two hours later, cells were subjected to western blotting and subsequent functional experiments. Cells used for this study were between passage 4 and 8.

### Real-time quantitative polymerase chain reaction (qPCR) analysis and Western blotting

FAP mRNA and protein expression was determined by qPCR and western blotting. Detailed methods can be found in Supplementary Data S1, available at *Rheumatology* online.

### Cell proliferation

Seventy-eight hours after transfection, cells (seeded on coverglass) were fixed with 4% paraformaldehyde for 20 min and permeabilized with 0.5% Triton X-100 for 30 min. The cells were then blocked with 2% BSA for 30 min and probed with an anti-Ki67 antibody (1:50) at 4°C overnight. After washing three times with Tris-buffered saline with Tween 20, they were incubated with secondary and tertiary antibodies (Supplementary Table S2, available at *Rheumatology* online) at room temperature for 1 h and 40 min, respectively. 4′,6-Diamidino-2-phenylindole (DAPI) solution was used to stain the cell nuclei. Proliferation rate was calculated as the ratio of Ki67 positive nuclei to DAPI staining. The experiment was conducted three times. Five visual fields were selected for each treatment, and the average value was used for statistical analysis.

### Statistical analysis

For the comparison of immunohistochemical (IHC) staining of different markers between GCA and control tissues, the Mann–Whitney *U*-test was performed, as the scores were not normally distributed. We compared the scores of GCA adventitia/media/intima *vs* control adventitia/media/intima, in both TAB and aorta. We further divided the intima of GCA-TAB into media–intima and inner-intima, and compared them in GCA-TAB. Values are presented as median and interquartile range (IQR).

For the *in vitro* study, independent experiments were done three times and for each experiment three to five replicates were included. One-way ANOVA was performed to compare the four groups. If significant, Student’s *t*-test was performed to compare the differences between two groups. Values are presented as the mean and standard error of mean (s.e.m.). Statistical analyses were performed using GraphPad Prism 9.0 software (GraphPad Software, Boston, MA, USA) and *P* < 0.05 was considered statistically significant.

## Results

### Patient characteristics

Patient characteristics are presented in Table 2. The diagnosis of GCA-related aorta aneurysm was made after the aorta surgery, based on the histopathological analysis, in all nine patients. All patients in the aorta-GCA group were in clinical remission (i.e. no GCA-related signs and symptoms) and six of them also underwent a fluorodeoxyglucose-PET-CT scan after the surgery, which did not show any clear signs of active vasculitis. Six of nine patients had not had any glucocorticoid or other immunosuppressive treatment in the past. One patient received glucocorticoid treatment 5 years before the aorta operation, duration of which is unknown (treated in a different hospital), one patient received, 8 years before the operation, glucocorticoid treatment lasting 2 years, and one patient received, 8 years before, glucocorticoid treatment lasting 1 year. We did not observe a special pattern in these three patients compared with the other six patients.

Regarding the disease duration, this is difficult to establish as by the time of symptoms or diagnosis there is already a chronic inflammation present in the vessel wall. Purely based on the symptom of headache, the disease duration for the cranial GCA (TAB) group was 2.7 (4) weeks (mean and s.d.), whereas for the aorta group this is not known for five patients (no symptoms and signs other than dyspnoea related to the aneurysm/secondary aorta valve insufficiency). For the remaining four patients, the disease duration was 4.8 (2.5) years (mean and s.d.), based on the time when the diagnosis of GCA was made, which was mostly a GCA with cranial complaints. Two of those four patients also had a positive TAB at the time of primary GCA diagnosis.

### Various fibroblast subtypes are detected in GCA-affected temporal arteries

**Figure 1.**
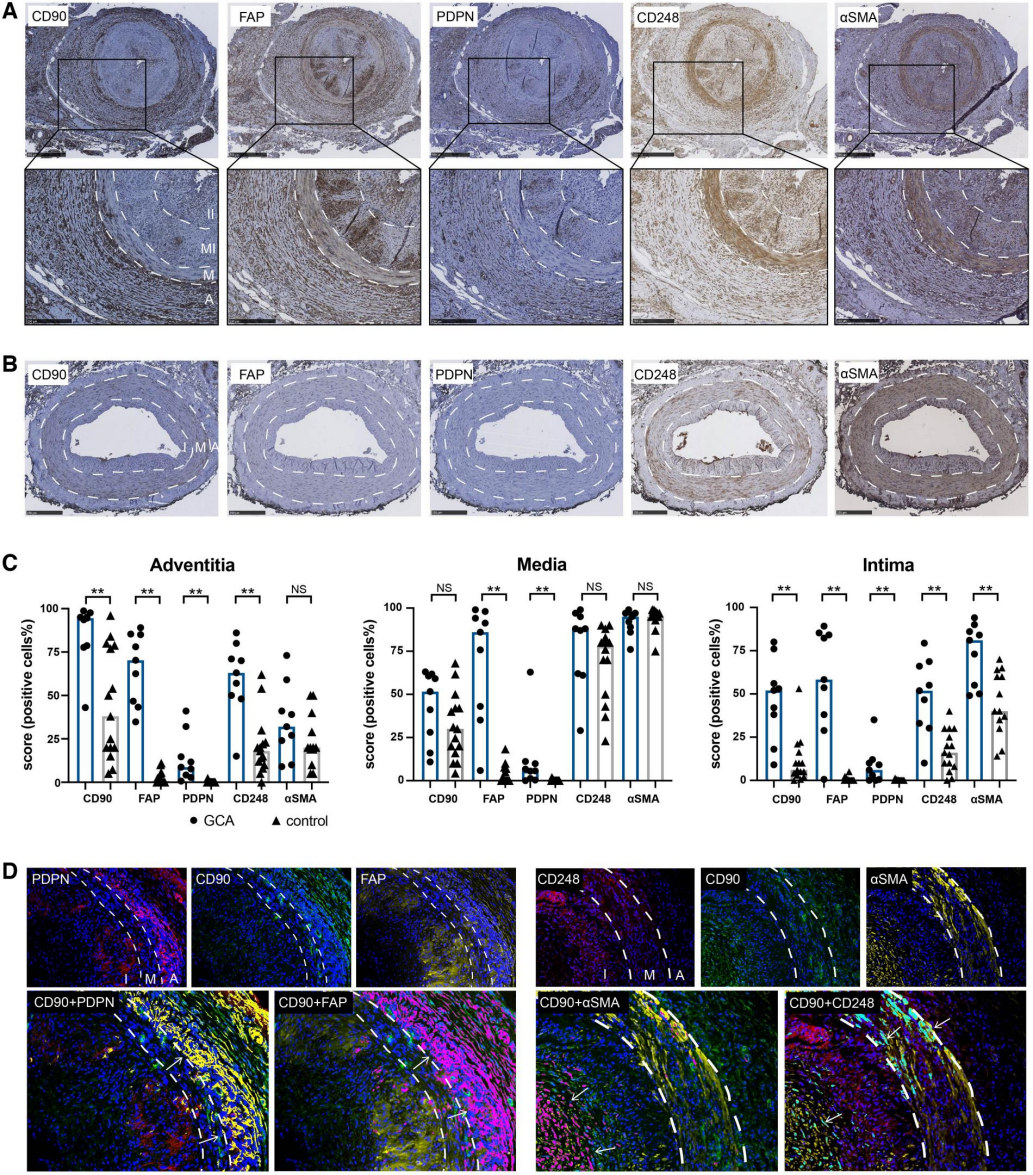
The expression of fibroblast phenotypic markers in temporal artery biopsies (TAB). (**A**) Representative picture of the immunohistochemical (IHC) staining of CD90, fibroblast activation protein α (FAP), podoplanin (PDPN), CD248 and α-smooth muscle actin (α-SMA) in giant cell arteritis (GCA)-affected TAB. (**B**) IHC staining in control TAB. (**C**) Scoring of fibroblast markers in adventitia, media, and intima (percentage of positive cells per layer). NS, not significant; *P < 0.05, **P < 0.01. (**D**) Triple immunofluorescence staining of CD90/FAP/PDPN and CD90/α-SMA/CD248 in GCA-affected TAB. PDPN, red; CD90, green; FAP, yellow; CD248, red; α-SMA, yellow; colocalization of CD90 and PDPN is shown in yellow, colocalization of CD90 and FAP is shown in magenta, colocalization of CD90 and αSMA is shown in magenta, colocalization of CD90 and CD248 is shown in cyan. Arrows indicate double positive areas. A, adventitia; M, media; I, intima. For GCA-TAB, intima includes media–intima (MI) and inner intima (II)

To identify fibroblast subtypes in GCA, we first stained TABs for fibroblast markers CD90, PDGFRA, FAP, PDPN, CD248 and α-SMA. Compared with controls, GCA-affected temporal arteries showed higher levels of CD90, FAP, PDPN and CD248 in the adventitia and intima (higher levels of CD90 and FAP in the media–intima and inner intima; higher levels of PDPN and CD248 in the media–intima), and isolated higher expression of α-SMA in the intima (both media–intima and inner intima) (Fig. 1A–C). PDGFRA was barely detected in TAB of either group despite positive staining in spleen tissue (Supplementary Fig. S3, available at *Rheumatology* online). To further investigate the distribution of various fibroblast subtypes, triple immunofluorescence staining for CD90/FAP/PDPN and CD90/α-SMA/CD248 was performed. We found that CD90^+^FAP^+^ activated fibroblasts and CD90^+^PDPN^+^ immunofibroblasts were predominantly located in the adventitia, whereas CD90^+^α-SMA^+^ myofibroblasts were observed mainly in the intima, and CD90^+^CD248^+^ pro-fibrotic fibroblasts were observed in the adventitia–media border and intima (Fig. 1D).

### Remodelling-associated growth factors are upregulated and spatially associated with intimal hyperplasia in GCA-affected temporal arteries

**Figure 2.**
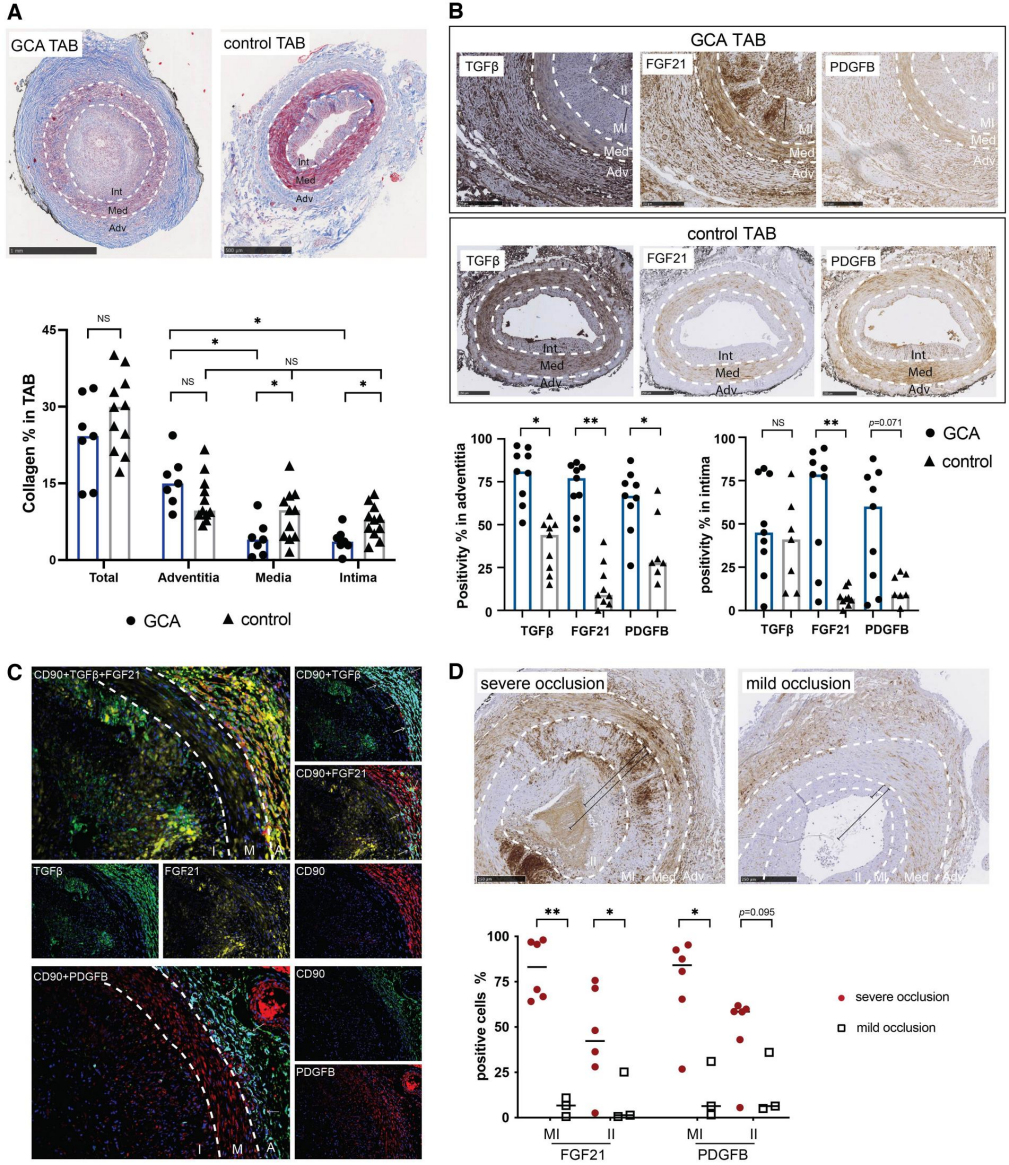
Expression of molecules related to vascular remodelling in temporal artery biopsies (TAB). (**A**) Masson trichrome staining showed no difference in the total collagen deposits (blue) between GCA and control TABs. However, reduced collagen content was found in media and intima of GCA compared with control TABs and compared with GCA adventitia. (**B**) Immunohistochemical staining showed higher expression of TGF-β, fibroblast growth factor 21 (FGF21) and platelet-derived growth factor B (PDGFB) in the adventitia of GCA-TAB than controls. FGF21 expression in intima was highly upregulated compared with control TAB. (**C**) Immunofluorescence staining of CD90 (red)/TGF-β (green)/FGF21 (yellow) and CD90 (green)/PDGFB (red) in GCA-positive TAB. Overlap of CD90 and TGF-β is shown in cyan (arrows). (**D**) FGF21 and PDGFB expression in media–intima, and FGF21 expression in the inner-intima was associated with intimal hyperplasia. *P < 0.05, **P < 0.01. Adv: adventitia; GCA: giant cell arteritis; Int: intima; the intima of GCA-TAB was further divided into media–intima (MI) and inner-intima (II); Med: media; NS: not significant

To explore tissue remodelling in temporal arteries, collagen content was assessed by Masson’s trichrome staining, and expression of TGF-β, FGF21 and PDGFB was assessed by IHC. Although the proportion of collagen in the whole vessel showed no significant differences between controls and GCA (30.0% *vs* 24.3%, *P* = 0.285), GCA-TAB showed significantly higher collagen proportions in the adventitia than in the media (15.0% *vs* 4.0%, *P* = 0.016) and intima (15.0% *vs* 3.6%, *P* = 0.016), while in control TAB collagen proportions were comparable in the three layers studied (adventitia 9.7%, media 9.8%, intima 7.9%) (Fig. 2A). In addition, GCA-affected TAB showed elevated expression of TGF-β (81.0% *vs* 44.0%, *P* < 0.001), PDGFB (66.9% *vs* 27.8%, *P* = 0.023) and FGF21 (77.1% *vs* 9.0%, *P* < 0.001) in the adventitia compared with controls (Fig. 2B). FGF21 was also strongly increased in the intima of GCA-affected TAB (78.5% *vs* 6.8%, *P* = 0.003), whereas TGF-β and PDGFB were not significantly different (Fig. 2B). The expression of these three markers was comparable in the media of GCA-TAB and control TAB (Supplementary Fig. S4B, available at *Rheumatology* online). To delineate the relationship between fibroblasts and vascular remodelling, immunofluorescence (IF) triple staining of CD90/FGF21/TGF-β and CD90/PDGFB was performed. Abundant co-localization of CD90 with TGF-β, FGF21 and PDGFB was observed in the adventitia (Fig. 2C): 54% of TGF-β expression colocalized with CD90 positivity, while 20% of FGF21 and PDGFB expression colocalized with CD90 positivity. Moreover, the extent of luminal occlusion was associated with FGF21 and PDGFB positivity in the media–intima region (Fig. 2D).

### Different distribution patterns of fibroblasts and remodelling-associated growth factors are observed in GCA-affected aorta tissue

**Figure 3.**
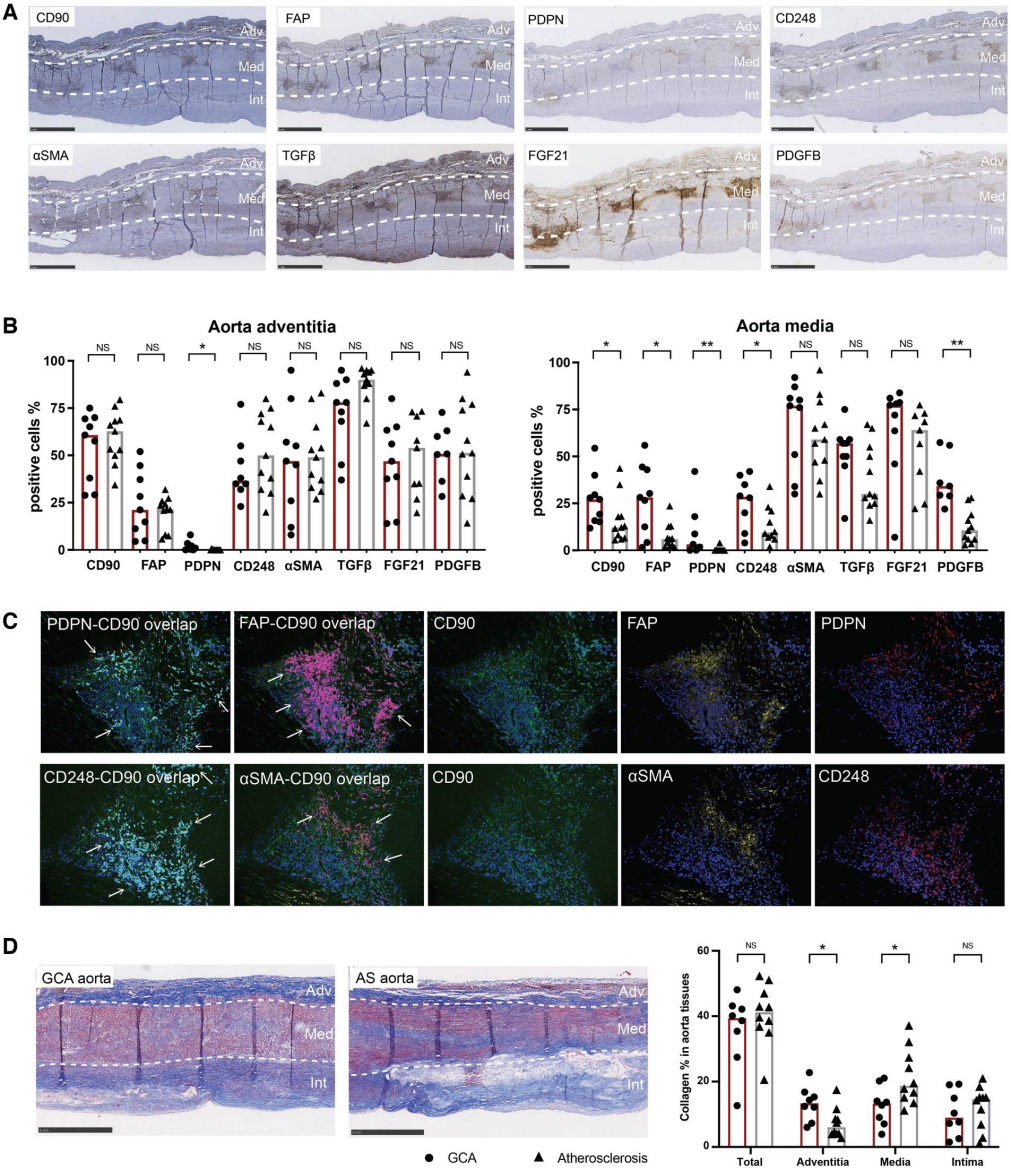
Fibroblast distribution patterns in aorta tissues. (**A**) Expression of fibroblast markers (CD90, fibroblast activation protein [FAP], podoplanin [PDPN], CD248, α-smooth muscle actin [α-SMA]) and cytokines related to remodelling (TGF-β, fibroblast growth factor 21 [FGF21], platelet-derived growth factor B [PDGFB]) in GCA-affected aorta tissues. Adv, adventitia; Med, media; Int, intima. (**B**) Scoring of fibroblast markers and cytokines related to remodelling in adventitia and media. NS, not significant; *P < 0.05, **P < 0.01. (**C**) Triple immunofluorescence staining of CD90/FAP/PDPN and CD90/α-SMA/CD248 in GCA-affected aorta tissues (structurally disrupted media). CD90, green; PDPN, red; CD24, red; FAP, yellow; α-SMA, yellow. Colocalization of CD90+CD248, CD90+PDPN is shown in cyan; colocalization of CD90+FAP, CD90+α-SMA is shown in magenta. Arrows indicate double positive areas. (**D**) Masson trichrome staining and collagen scoring in aorta tissues. NS: not significant; *P < 0.05

Different expression patterns of fibroblast markers and growth factors were found in GCA-affected aortas when compared with TABs. GCA-affected aorta tissues showed increased expression of CD90, FAP, PDPN, CD248 and PDGFB in the media compared with atherosclerotic aorta used as control tissue, whereas α-SMA, TGF-β and FGF21 expression in the media showed no significant differences (Fig. 3A, B, Supplementary Fig. S4C, available at *Rheumatology* online). In addition, we observed slightly higher expression of PDPN (1.0% *vs* 0%, *P* = 0.015) in the adventitia of the GCA-affected aorta and higher expression of FAP (2.6% *vs* 13.1%, *P* = 0.024) in the intima of the atherosclerotic aorta (Fig. 3A, B; Supplementary Fig. S4D, available at *Rheumatology* online). Similar to TAB, PDGFRA was barely detected in aorta tissues (Supplementary Fig. S3, available at *Rheumatology* online). To study the distribution of fibroblast subtypes, triple IF staining was performed for CD90/FAP/PDPN and CD90/α-SMA/CD248. CD90^+^FAP^+^, CD90^+^PDPN^+^ and CD90^+^CD248^+^ fibroblasts were predominantly located in structurally disrupted media in GCA-affected aorta (Fig. 3C). Although some CD90^+^α-SMA^+^ myofibroblasts were also present in structurally disrupted media (Fig. 3C), this subtype was located mainly in the intima (Supplementary Fig. S4E, available at *Rheumatology* online).

We also observed different collagen deposition patterns in the aorta tissues compared with those in TAB. Although there were no significant differences in overall collagen deposits between GCA- and atherosclerosis-aorta tissues (36.0% *vs* 40.7% of the whole vessel areas, *P* = 0.641), we did observe higher adventitial collagen deposits (13.4% *vs* 7.3%, *P* = 0.027) and lower media collagen deposits (12.7% *vs* 21.5%, *P* = 0.043) in GCA affected aorta tissues compared with GCA-atherosclerosis aorta (Fig. 3D).

### CD90^+^FAP^+^ fibroblasts show a proliferative phenotype

**Figure 4.**
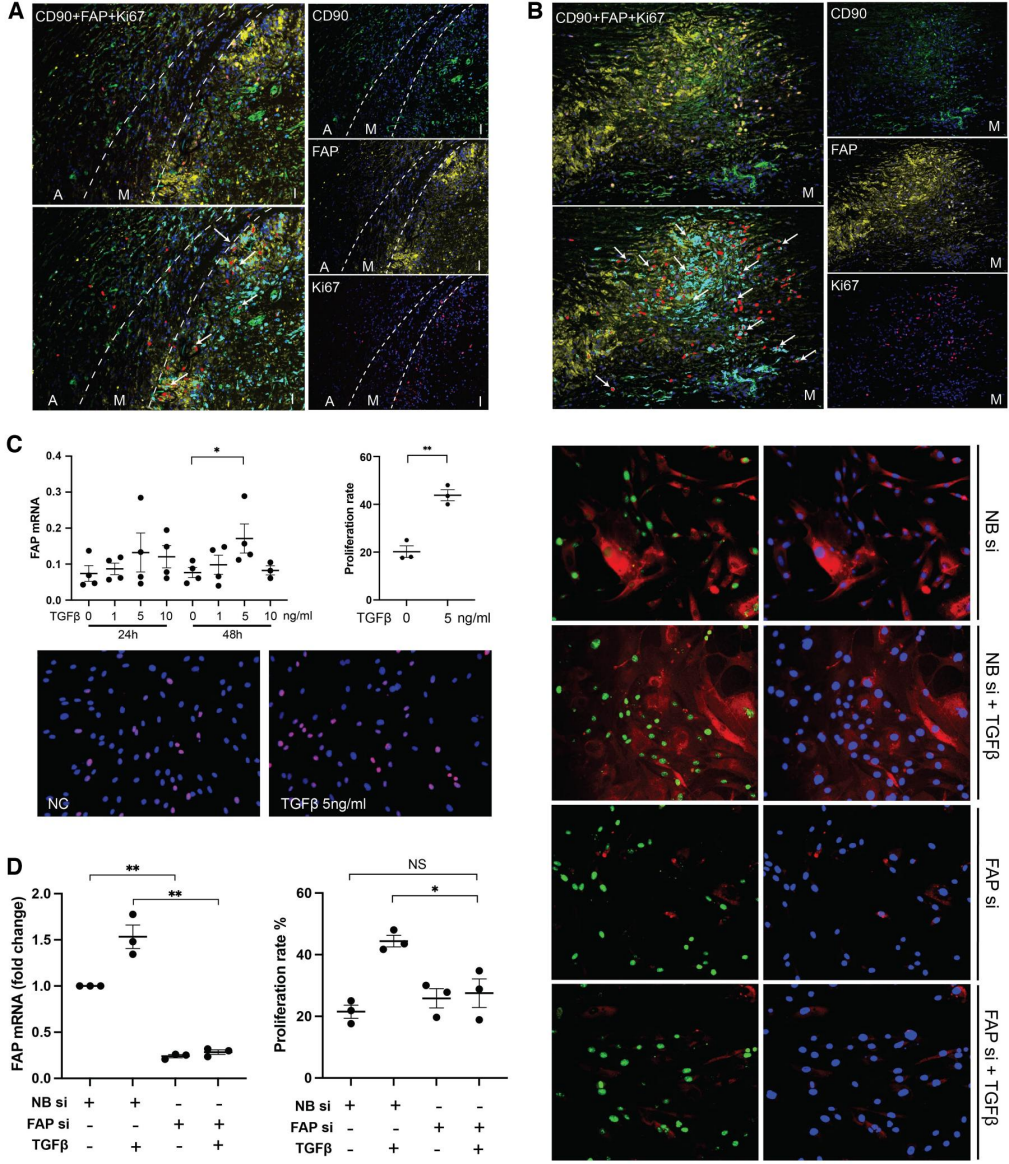
The relationship between fibroblast activating protein (FAP) and human aortic fibroblast proliferation. (**A**, **B**) Triple immunofluorescent staining of CD90/FAP/Ki67 in GCA-affected temporal artery (**A**) and aorta (structurally disrupted media) (**B**). Ki67, red; CD90, green; FAP, yellow; colocalization of CD90/FAP, cyan; arrows indicate CD90^+^FAP^+^Ki67^+^ cells. A, adventitia; M, media; I, intima; GCA, giant cell arteritis. (**C**) TGF-β1 5 ng/ml 48 h promoted mRNA expression of FAP and proliferation in human aortic adventitial fibroblasts (HAoAF). Proliferation rate was calculated as Ki67 positive nuclei divided by 4′,6-diamidino-2-phenylindole positive nuclei; Ki67, red. (**D**) HAoAF were transfected with small interfering RNA (siRNA) targeting FAP, and were treated with TGF-β1 6 h later. After 24 h, cells were collected for qPCR; 72 h later, cells were collected for Ki67 staining. FAP knockdown decreased the effect of TGF-β1 on cell proliferation. FAP, red; Ki67, green. NB si: non-binding small interfering RNA; FAP siRNA: small interfering RNA targeting FAP. NS: not significant; *P < 0.05, **P < 0.01

To investigate the proliferation of fibroblasts at the site of inflammation in GCA, IF staining of Ki67 was performed in GCA-affected TAB (Fig. 4A) and aortic tissues (Fig. 4B). Among all proliferative fibroblasts (CD90^+^Ki67^+^), ∼80% were FAP positive in GCA-affected TAB and aorta tissues. In addition, we observed a small population of CD90^+^PDPN^+^ and CD90^+^CD248^+^ proliferating fibroblasts (<15%) in GCA-affected TAB (Supplementary Fig. S5A–C, available at *Rheumatology* online).

### FAP knockdown suppresses TGF-β-induced proliferation of fibroblasts

Given the preponderance of FAP positivity among proliferating fibroblasts, we further investigated the role of FAP in fibroblast proliferation *in vitro*. First, to induce FAP expression, HAoAF were cultured in the presence of several concentrations of TNFα (5, 20 and 100 ng/ml) or TGF-β1 (1, 5 and 10 ng/ml). TNF-α upregulated IL-6 but had no effect on FAP expression in HAoAF (Supplementary Fig. S6A, available at *Rheumatology* online). In contrast, TGF-β1 showed no effect on IL-6, but it did promote mRNA and protein expression of FAP as well as proliferation of HAoAF cells (optimal concentration 5 ng/ml at 48 h) (Fig. 4C, Supplementary Fig. S6B, C, available at *Rheumatology* online). To further investigate whether FAP contributes to fibroblast proliferation, FAP knockdown was induced by FAP siRNA transfection. FAP knockdown cells showed 80–90% reduced FAP expression at both the mRNA and protein level (Fig. 4D, Supplementary Fig. S6D–F, available at *Rheumatology* online). There were no significant differences in the mRNA levels of CD248, α-SMA and podoplanin at the end of the cell culture (72 h) between the groups (Supplementary Fig. S6G, available at *Rheumatology* online). FAP knockdown abolished TGF-β1 induced proliferation, as the proliferation rate returned to that of TGF-β1 unstimulated fibroblasts (27.5% *vs* 44.4%, *P* = 0.0278, unstimulated: 21.5%) (Fig. 4D).

## Discussion

In this study, we comprehensively describe the fibroblast landscape and heterogeneity in the arteries of patients with GCA based on sets of phenotypic and functional markers. We document different patterns of fibroblast subtypes in temporal artery and aorta. In addition, we show the presence of proliferating fibroblasts at the sites of vascular remodelling in GCA, a process seemingly related to expression of FAP.

Using CD90 as pan-marker of fibroblasts, we identified four fibroblast subtypes in both TAB and aorta: FAP^+^ activated fibroblasts, PDPN^+^ immunofibroblasts, CD248^+^ pro-fibrotic fibroblasts and α-SMA^+^ myofibroblasts. We found different distribution patterns of fibroblast subtypes in TAB and aorta tissues. Although the role played by fibroblasts in GCA remains to be further elucidated, this differential expression of various fibroblast cell surface markers may be related to different disease stages (i.e. early in TAB and late in the aorta aneurysm) of the vasculopathy process in different vascular beds (i.e. vascular thickening/stenosis in temporal arteries *vs* vascular dilatation/aneurysm formation in aorta).

The remodelling pattern in GCA is different from that with Takayasu arteritis (TAK). TAK is characterized by extensive arterial wall fibrosis mediated by ECM production from adventitial fibroblasts [21]. It is reported that the expression of collagen 1, collagen 3, fibronectin, α-SMA and TGF-β in the adventitia of TAK aorta is significantly higher than in normal arteries [22, 23]. In contrast, our data showed elevated TGF-β levels in the adventitia of GCA-TAB compared with controls, but not in GCA aortas. We observed no differences in α-SMA levels in GCA-affected arteries compared with control arteries. Despite thickening of the adventitia and intima in GCA-TAB, the total collagen deposits did not differ between GCA and control arteries. This suggests that the cellular component (i.e. proliferation) is more important for vascular thickening than increased collagen production in GCA vasculopathy.

Interactions between fibroblasts and immune cells have been reported as playing an important role in the pathogenesis of several chronic autoimmune diseases, such as rheumatoid arthritis, Sjögren’s syndrome and systemic sclerosis [13, 24, 25]. PDPN expressing stromal cells are defined as immune-modulatory fibroblasts or ‘immunofibroblasts’. Deletion of the PDPN^+^ immunofibroblasts prevented the formation of tertiary lymphoid structures and reduced local immune pathology in Sjögren’s syndrome [13, 26]. More recently, fibroblast–lymphocyte interaction has also received increasing attention in GCA. Greigert *et al.* described a positive feedback loop by which myofibroblasts (CD90^+^α-SMA^+^MYH11^+^) located in the neointima of GCA-affected temporal arteries produce Th1/Th17 polarizing cytokines (IL-12/IL-23) in response to IFN-γ and TNF-α, thereby sustaining T cell polarization [27]. This study suggests a mechanism by which fibroblasts contribute to the perpetuation of the inflammatory process in vascular lesions through interactions with the T cells. Whether and how fibroblasts interact with other inflammatory cells in GCA remains to be investigated.

Considering the possible role of vascular fibroblasts in chronic inflammation and remodelling, targeting pathological processes mediated by fibroblasts may represent a novel therapeutic approach in GCA. In this study, the proliferation of FAP^+^ fibroblasts may be related to vascular thickening and occlusion. Depletion of FAP^+^ fibroblasts may prevent pathological remodelling in GCA-affected vessels. Currently, targeted imaging of FAP and FAP-targeted therapy, are gaining tremendous interest in cancer and inflammatory diseases [28]. Unexpected positivity of large arteries with FAP inhibitor (FAPI)-based radiotracers, such as [^68^Ga]FAPI, has been reported in clinically quiescent GCA patients [29]. One case report showed successful visualization of aortic and arterial inflammation in a patient suffering from GCA using [^68^Ga]FAPI-04-PET [30]. This supports the potential of FAP targeted imaging for visualization of vascular inflammation in GCA warranting further investigation especially in comparison with the current gold standard [^18^F]fluorodeoxyglucose-PET. Recent advancements in therapeutic targeting of cancer-associated fibroblasts suggests that this approach may also be applicable in the future for patients with GCA [31].

The primary strength of our study lies in the thorough immunohistochemical analysis of diverse fibroblast markers. This analysis represents the first step in identifying and characterizing the phenotype and functional role of fibroblast subtypes in GCA. Furthermore, we employed an *in vitro* model using human adventitial fibroblasts to modulate and inhibit FAP expression, providing valuable functional insights into the role of FAP in GCA pathogenesis. However, a limitation of our study is the relatively small sample size of patients requiring validation and follow-up studies. In our study, the patients with a GCA-related aortic aneurysm being operated were younger than the patients with cranial GCA (TAB positive). In line with our data, it has been reported that patients with involvement of the aorta (and thus at risk for development of an aorta aneurysm) are younger than those with cranial GCA [32–34]. While patients with cranial GCA showed high CRP/ESR levels, the low CRP/ESR in patients with GCA at the time of aorta surgery may reflect persistent pathological remodelling in aorta tissues rather than clinically manifested disease activity. A previous study by Maleszewski *et al.* also showed persistence of an inflammatory infiltrate in the temporal artery up to 1 year follow-up in patients with GCA clinically and biochemically in remission [35]. The past treatment with glucocorticoids or other immunosuppressive medication may influence the histology of the artery. However, we did not observe a special pattern in the three patients with GCA-related aorta aneurysm that had been treated with glucocorticoids in the past compared with the other six patients. Future research should further clarify the exact meaning of the persisting inflammatory infiltrate in the arteries affected by GCA and whether fibroblast subtypes differ across disease stages (treatment, remission, relapse, etc.).

In conclusion, our study demonstrates that distinct fibroblast subtypes are present in blood vessels affected by GCA. The varying patterns observed in the temporal artery and aorta suggest different pathogenic mechanisms based on vascular structure and disease duration. The proliferation of adventitial fibroblasts is predominantly dependent on FAP expression. Further research is essential to elucidate the diverse phenotypic and functional subtypes of fibroblasts in GCA vasculopathy. Specifically, integrating spatial transcriptomics with *in vitro* functional studies could lead to the identification of pathological and reparative/healing fibroblast populations, which might be targeted to develop novel treatments for GCA patients.

## Supplementary Material

keaf143_Supplementary_Data

## Data Availability

Data are available upon reasonable request.
