# Supplementary material for: Distinct landscapes of fibroblast subtypes in arteries of patients with giant cell arteritis
Source: Rheumatology (Oxford). 2025 Mar 14;64(7):4382–92. doi: 10.1093/rheumatology/keaf143 (PMC12212911; doi:10.1093/rheumatology/keaf143)
Supplement: keaf143_Supplementary_Data [file keaf143_supplementary_data.zip › keaf143_Supplementary_Data/rhe-24-2584-File007.docx]

**Supplementary Tables**

Supplementary Table S1. Antibodies used for immunohistochemistry (IHC) staining

| Target | Antigen retrieval | Manufacturer & code | Host & isotype | Dilution | Secondary antibody | Dilution |
| --- | --- | --- | --- | --- | --- | --- |
| FAP | pH 9 | abcam ab207178 | Rabbit mono IgG | 1:150 | R&D VC003 | undiluted |
| CD90 | pH 9 | NOVUS NBP2-37330 | Mouse IgG1 | 1:400 | DAKO Envision K4001 | undiluted |
| PDPN | pH 9 | DAKO M361929-2 | Mouse IgG1 | 1:40 | DAKO Envision K4001 | undiluted |
| CD248 | pH 9 | Invitrogen MA5-26838 | Mouse IgG1 | 1:75 | DAKO Envision K4001 | undiluted |
| α-SMA | pH 9 | abcam ab124964 | Rabbit mono IgG | 1:1000 | DAKO P448 | 1:50 |
| TGFβ | pH 9 | Biorad MAC797 | Mouse IgG1 | 1:1000 | DAKO P260 | 1:50 |
| FGF21 | pH 7.4 | Abclonal A3908 | Rabbit mono IgG | 1:75 | R&D VC003 | undiluted |
| PDGFB | pH 6 | abcam ab23914 | Rabbit poly | 1:75 | R&D VC003 | undiluted |
| PDGFRA | pH 9 | abcam ab203491 | Rabbit mono IgG | 1:50 | R&D VC003 | undiluted |

Supplementary Table S2. Antibodies used for immunofluorescence (IF) staining

(1) Protocols of antibodies for immunofluorescence staining

| Target | Antigen retrieval | Manufacturer & code | Host & isotype | Dilution |
| --- | --- | --- | --- | --- |
| CD90 | pH 9 | NOVUS, NBP2-37330 | Mouse IgG1 | 1:200 |
| CD90 | pH 9 | Proteintech, 66766-1-Ig | Mouse IgG2a | 1:75 |
| FAP | pH 9 | abcam, ab207178 | Rabbit mono IgG | 1:100 |
| PDPN | pH 9 | DAKO, M361929-2 | Mouse IgG1 | 1:40 |
| α-SMA | pH 9 | abcam, ab124964 | Rabbit mono IgG | 1:1000 |
| CD248 | pH 9 | Invitrogen MA5-26838 | Mouse IgG1 | 1:75 |
| Vimentin | pH 9 | Abclonal, A19607 | Rabbit IgG | 1:200 |
| Ki67 | pH 9 | DAKO, M724029 | Mouse IgG1 | 1:50 |
| FGF21 | pH 9 | Abclonal A3908 | Rabbit mono IgG | 1:75 |
| TGF-β | pH 9 | Biorad MAC797 | Mouse IgG1 | 1:1000 |
| PDGFB | pH 9 | abcam ab23914 | Rabbit poly | 1:50 |
| Ki67 | pH 9 | abcam, ab16667 | Rabbit mono IgG | 1:200 |

(2) Double/triple staining patterns

CD90/PDPN/FAP

|  | CD90 (Proteintech) | PDPN | FAP (abcam, ab207178) | Nucleus |
| --- | --- | --- | --- | --- |
| Primary ab | Mouse IgG2a, 1:75 | Mouse IgG1, 1:40 | Rabbit IgG, 1:100 |  |
| Secondary ab | Rat anti-mouse IgG2a (Biolegend, RMG2a-1)  1:40 | Goat anti-mouse IgG1 (Southern Biotech)  1:50 |  |  |
| Tertiary ab | Donkey anti-rat IgG  (abcam, ab150153)  1:50 | Donkey anti-goat IgG  (abcam, ab175704)  1:50 | Donkey anti-rabbit IgG  (abcam, ab150075)  1:50 |  |
| Conjugate/dye | AF488 | AF568 | AF647 | DAPI |

CD90/CD248/αSMA

|  | CD90 (Proteintech) | CD248 | αSMA | Nucleus |
| --- | --- | --- | --- | --- |
| Primary ab | Mouse IgG2a, 1:75 | Mouse IgG1, 1:75 | Rabbit IgG, 1:1000 |  |
| Secondary ab | Rat anti-mouse IgG2a (Biolegend, RMG2a-1)  1:40 | Goat anti-mouse IgG1 (Southern Biotech)  1:50 |  |  |
| Tertiary ab | Donkey anti-rat IgG  (abcam, ab150153)  1:50 | Donkey anti-goat IgG  (abcam, ab175704)  1:50 | Donkey anti-rabbit IgG  (abcam, ab150075)  1:50 |  |
| Conjugate/dye | AF488 | AF568 | AF647 | DAPI |

CD90/TGFβ/FGF21

|  | CD90 (Proteintech) | TGFβ | FGF21 | Nucleus |
| --- | --- | --- | --- | --- |
| Primary ab | Mouse IgG2a, 1:75 | Mouse IgG1, 1:1500 | Rabbit IgG, 1:60 |  |
| Secondary ab | Rat anti-mouse IgG2a (Biolegend, RMG2a-1)  1:40 | Goat anti-mouse IgG1 (Southern Biotech)  1:50 |  |  |
| Tertiary ab | Donkey anti-rat IgG  (abcam, ab175475)  1:50 | Donkey anti-goat IgG  (abcam, ab150129)  1:50 | Donkey anti-rabbit IgG  (abcam, ab150075)  1:50 |  |
| Conjugate/dye | AF568 | AF488 | AF647 | DAPI |

CD90/Ki67/FAP

|  | CD90 (Proteintech) | Ki67 | FAP | Nucleus |
| --- | --- | --- | --- | --- |
| Primary ab | Mouse IgG2a, 1:75 | Mouse IgG1, 1:50 | Rabbit IgG, 1:100 |  |
| Secondary ab | \| Rat anti-mouse IgG2a (Biolegend, RMG2a-1)  1:50 \| \| --- \| | \| Goat anti-mouse IgG1 (Southern Biotech)  1:50 \| \| --- \| |  |  |
| Tertiary ab | \| Donkey anti-rat IgG  (abcam, ab150155)  1:50 \| \| --- \| | \| Donkey anti-goat IgG  (abcam, ab175704)  1:50 \| \| --- \| | \| Donkey anti-rabbit IgG  (abcam, ab150075)  1:50 \| \| --- \| |  |
| Conjugate/dye | AF488 | AF568 | AF647 | DAPI |

CD90/Ki67/PDPN

|  | CD90 (Proteintech) | PDPN | Ki67 (abcam) | Nucleus |
| --- | --- | --- | --- | --- |
| Primary ab | Mouse IgG2a, 1:75 | Mouse IgG1, 1:40 | Rabbit IgG, 1:200 |  |
| Secondary ab | \| Rat anti-mouse IgG2a (Biolegend, RMG2a-1)  1:50 \| \| --- \| | \| Goat anti-mouse IgG1 (Southern Biotech)  1:50 \| \| --- \| |  |  |
| Tertiary ab | \| Donkey anti-rat IgG  (abcam, ab150155)  1:50 \| \| --- \| | \| Donkey anti-goat IgG  (abcam, ab150129)  1:50 \| \| --- \| | \| Donkey anti-rabbit IgG  (abcam, ab175470)  1:50 \| \| --- \| |  |
| Conjugate/dye | AF647 | AF488 | AF568 | DAPI |

CD90/Ki67/CD248

|  | CD90 (Proteintech) | CD248 | Ki67 | Nucleus |
| --- | --- | --- | --- | --- |
| Primary ab | Mouse IgG2a, 1:75 | Mouse IgG1, 1:75 | Rabbit IgG, 1:200 |  |
| Secondary ab | \| Rat anti-mouse IgG2a (Biolegend, RMG2a-1)  1:50 \| \| --- \| | \| Goat anti-mouse IgG1 (Southern Biotech)  1:50 \| \| --- \| |  |  |
| Tertiary ab | \| Donkey anti-rat IgG  (abcam, ab150155)  1:50 \| \| --- \| | \| Donkey anti-goat IgG  (abcam, ab150129)  1:50 \| \| --- \| | \| Donkey anti-rabbit IgG  (abcam, ab175470)  1:50 \| \| --- \| |  |
| Conjugate/dye | AF647 | AF488 | AF568 | DAPI |

Vimentin/Ki67

|  | Ki67 | Vimentin | Nucleus |
| --- | --- | --- | --- |
| Primary ab | Mouse IgG1, 1:50 | Rabbit IgG, 1:200 |  |
| Secondary ab | Goat anti-mouse IgG1 (Southern Bioteh)  1:50 |  |  |
| Tertiary ab | Donkey anti-goat IgG  (abcam, ab150129)  1:50 | Donkey anti-rabbit IgG  (abcam, ab150075)  1:50 |  |
| Conjugate/dye | AF568 | AF647 | DAPI |

CD90/PDGFB

|  | CD90 (NOVUS) | PDGFB | Nucleus |
| --- | --- | --- | --- |
| Primary ab | Mouse IgG1, 1:200 | Rabbit IgG, 1:60 |  |
| Secondary ab | Goat anti-mouse IgG1 (Southern Bioteh)  1:50 |  |  |
| Tertiary ab | Donkey anti-goat IgG  (abcam, ab150129)  1:50 | Donkey anti-rabbit IgG  (abcam, ab150075)  1:50 |  |
| Conjugate/dye | AF488 | AF568 | DAPI |

Ki67 staining in cells (with FAP)

|  | Ki67 | FAP | Nucleus |
| --- | --- | --- | --- |
| Primary ab | Mouse IgG1, 1:50 | Rabbit IgG, 1:100 |  |
| Secondary ab | \| Goat anti-mouse IgG1 (Southern Biotech)  1:50 \| \| --- \| |  |  |
| Tertiary ab | \| Donkey anti-goat IgG  (abcam, ab150129)  1:50 \| \| --- \| | \| Donkey anti-rabbit IgG  (abcam, ab175470)  1:50 \| \| --- \| |  |
| Conjugate/dye | AF488 | AF568 | DAPI |

Supplementary Table S3. Antibodies for western blotting

| Target | Manufacturer & code | Host & isotype | Dilution | Secondary antibody | Dilution |
| --- | --- | --- | --- | --- | --- |
| FAP | abcam ab207178 | Rabbit mono IgG | 1:1000 | IRDye® 680CW goat anti-rabbit R&D | 1:5000 |
| α-SMA | abcam ab124964 | Rabbit mono IgG | 1:10000 | IRDye® 680CW goat anti-rabbit DAKO | 1:5000 |
| GAPDH | ABclonal AC033 | Mouse mono IgG | 1:20000 | IRDye® 800CW goat anti-mouse IgG | 1:5000 |

**Supplementary Figures**

**
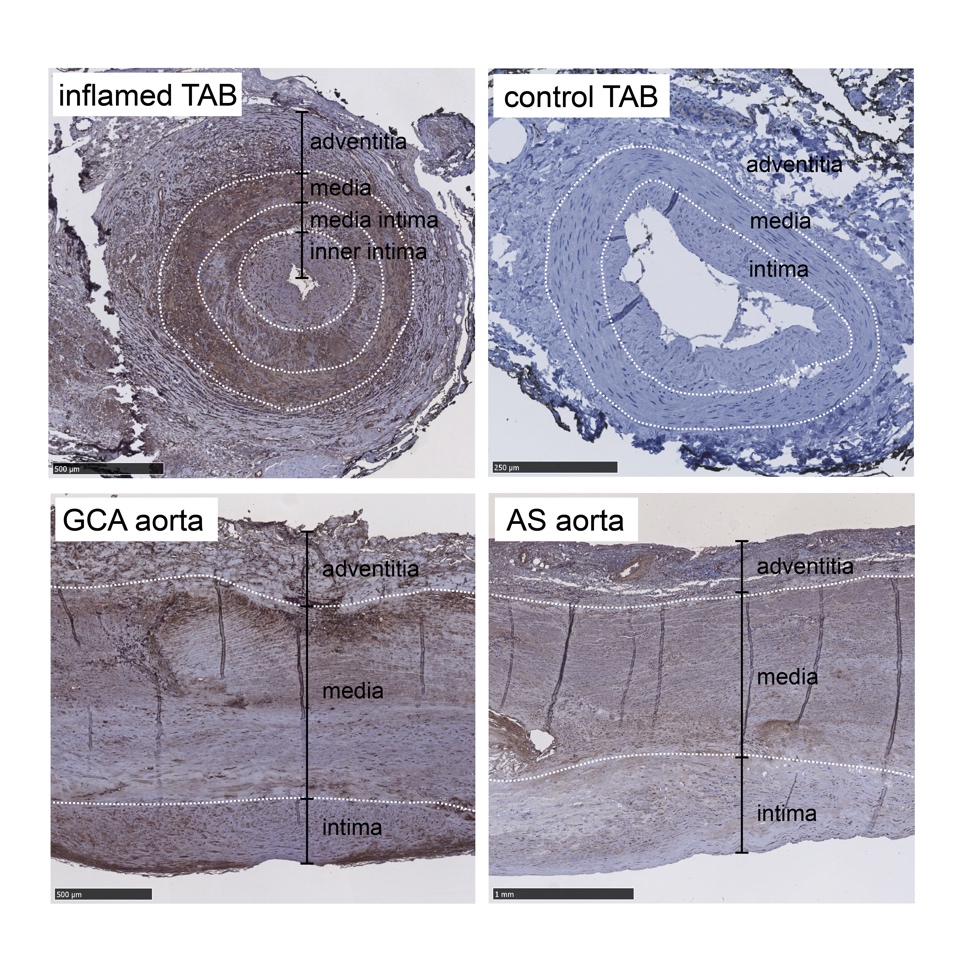
**

**Supplementary Figure S1.** Tissue topology of temporal artery and aorta.

*
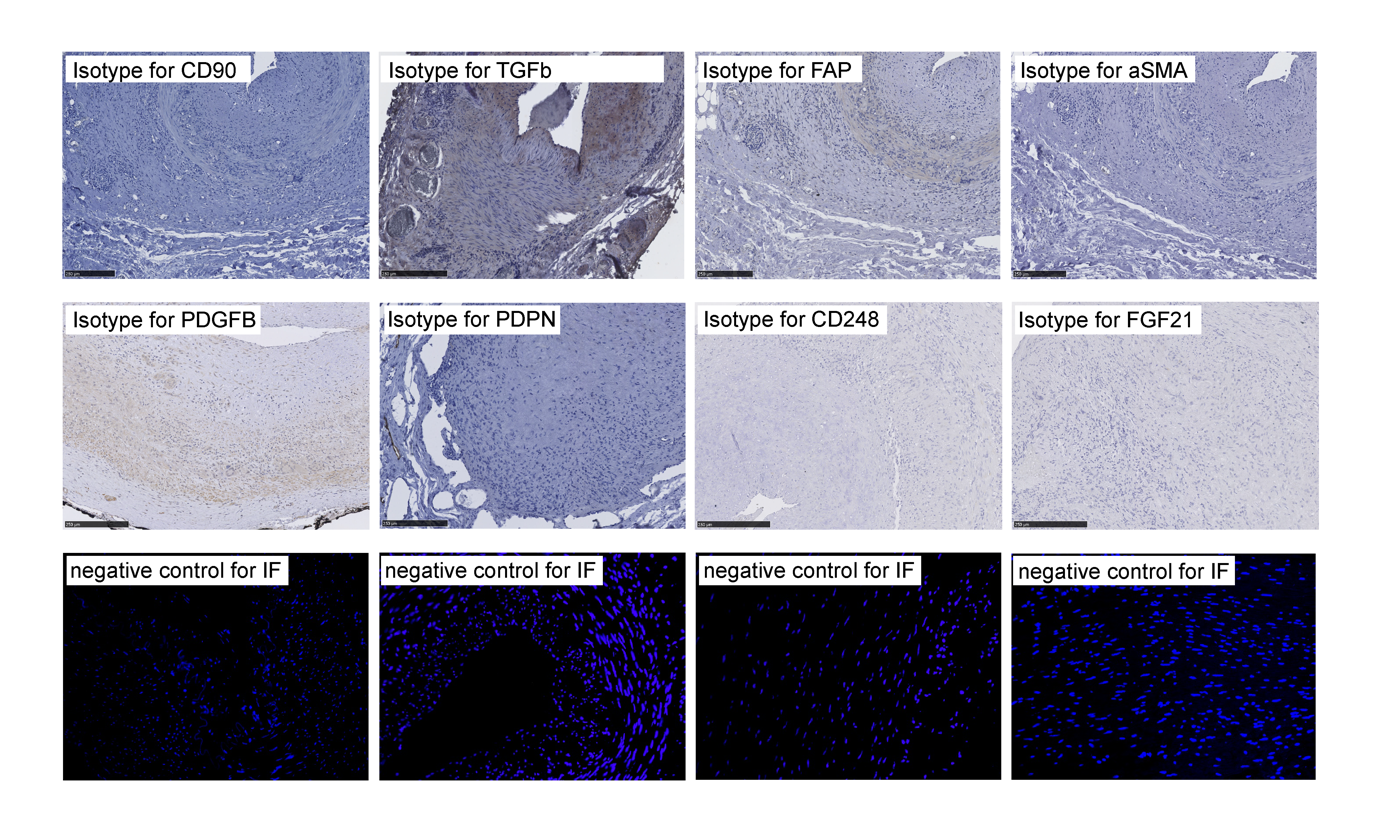
*

**Supplementary Figure S2.** Isotype control for IHC staining and negative control for immunofluorescence staining.

**
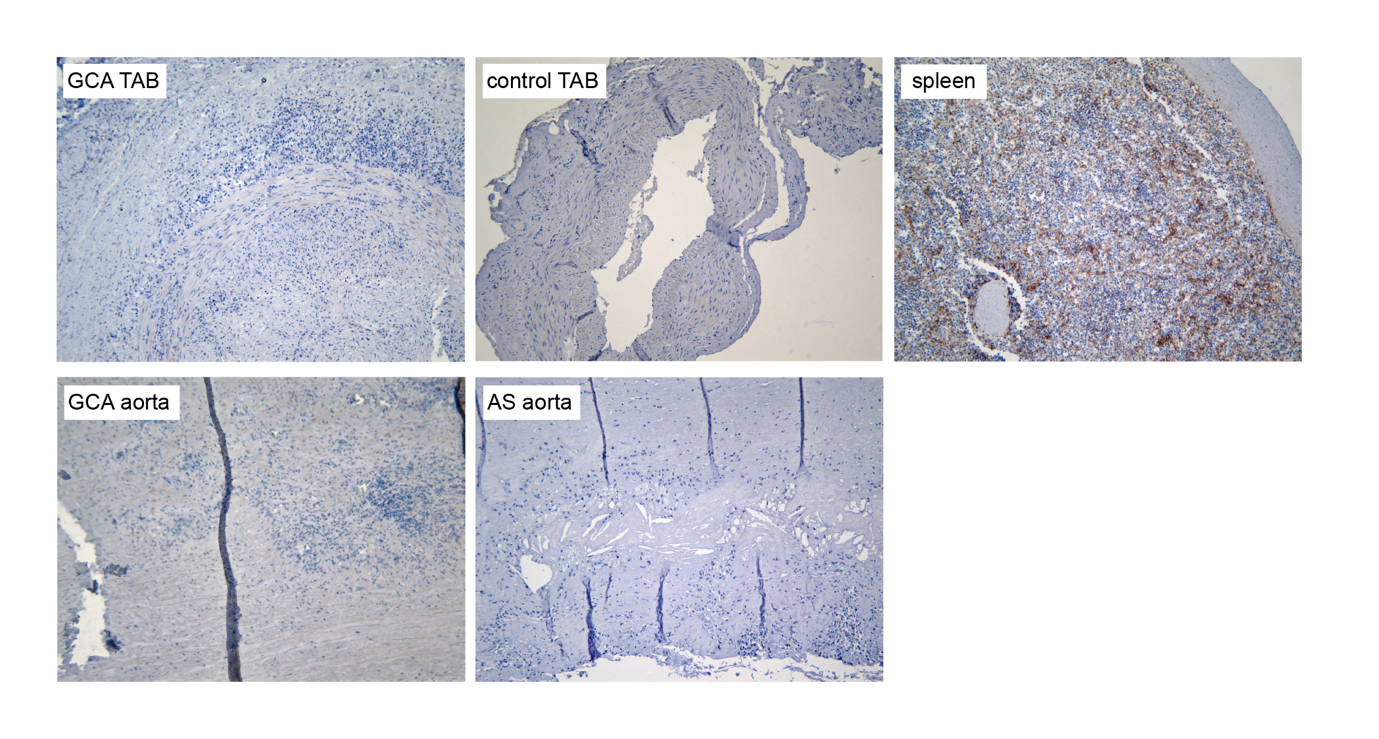
**

**Supplementary Figure S3**. Representative pictures of the immunohistochemical staining of PDGFRA in temporal artery biopsy (TAB) and aorta tissues showing the lack of PDGFRA expression by any cell type, including fibroblasts. TAB: temporal artery biopsy; GCA: giant cell arteritis; AS: atherosclerosis. Staining in spleen tissue as positive control.


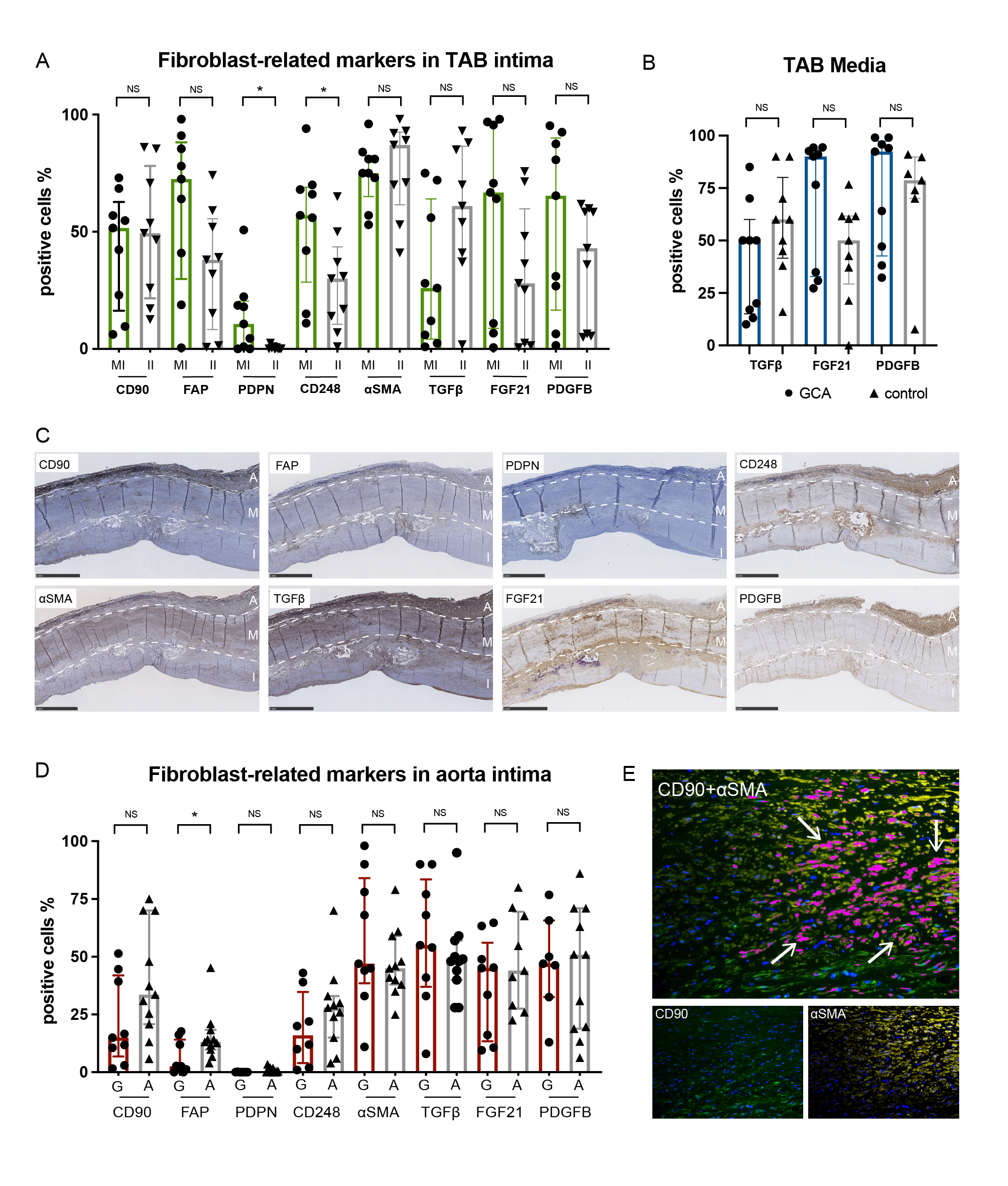


**Supplementary Figure S4**. Additional staining and analysis of fibroblast related markers in temporal arteries and aorta tissues. (A) Scoring of fibroblast markers and cytokines in media-intima (MI) and inner intima (II) of GCA-affected temporal arteries. (B) Scoring of cytokines in the media of GCA-affected TAB vs control TAB. (C) Fibroblast markers and cytokines in atherosclerotic aorta tissues. (D) Expression of fibroblast markers and growth factors in the intima of aorta tissues, shown as percentage of positive cells. A=atherosclerosis; G=giant cell arteritis; (E) Double staining of CD90/α-SMA in GCA-affected aorta tissues (intima). CD90-green, αSMA-yellow, colocalization of CD90 and α-SMA-magenta (arrows). NS=not significant, *p<0.05.

*
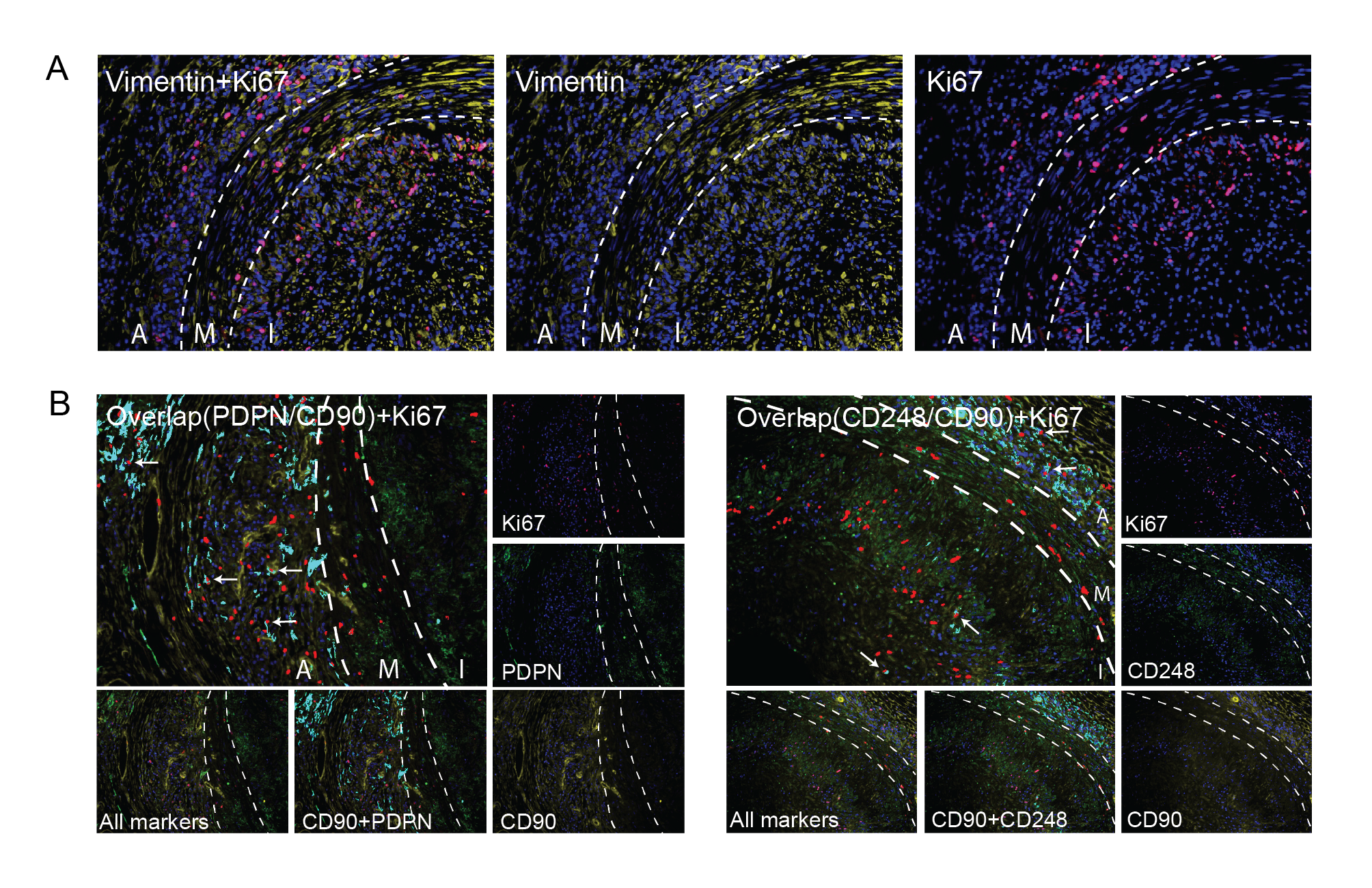
*

**Supplementary Figure S5**. Immunofluorescence staining of Vimentin/Ki67 (A), CD90/PDPN/Ki67 (B), and CD90/CD248/Ki67 (C) in GCA-affected temporal artery. Ki67 positive areas showed red nucleus, colocalization areas of CD90/PDPN and CD90/CD248 showed cyan. A-adventitia, M-media, I-intima. Arrows indicates triple positive cells.

*
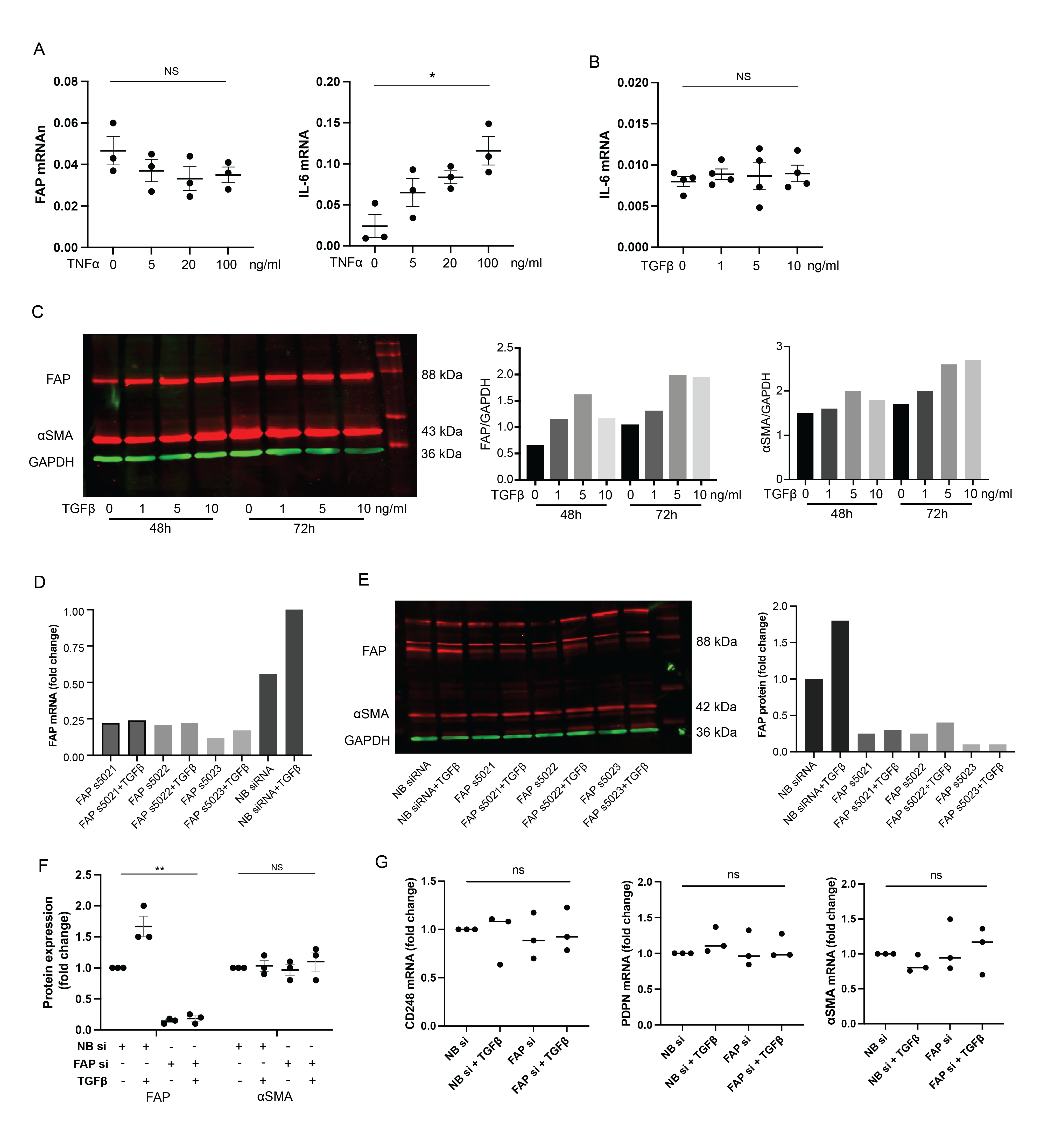
*

**Supplementary Figure S6**. In vitro experiments. (A) mRNA relative expression of FAP and IL-6 in human aortic adventitial fibroblasts (HAoAF), with the stimulation of TNFα (5, 20, 100ng/ml) for 24h. (B) mRNA relative expression of IL-6 in HAoAF, with the stimulation of TGFβ (1, 5, 10ng/ml) for 48h. (C) Western blotting (WB) confirmed the regulation of TGFβ on FAP protein levels. (D, E) FAP siRNA s5023 had the best knockdown efficacy, as shown by qPCR and WB. (F) Protein levels of FAP and αSMA 78h after FAP siRNA transfection. (G) mRNA expression of CD248, podoplanin (PDPN), αSMA 78 hours after FAP siRNA transfection. NS=not significant, *p<0.05, **p<0.01.
