# Supplementary material for: Distinct landscapes of fibroblast subtypes in arteries of patients with giant cell arteritis
Source: Rheumatology (Oxford). 2025 Mar 14;64(7):4382–92. doi: 10.1093/rheumatology/keaf143 (PMC12212911; doi:10.1093/rheumatology/keaf143)
Supplement: keaf143_Supplementary_Data [file keaf143_supplementary_data.zip › keaf143_Supplementary_Data/rhe-24-2584-File008.docx]

**Supplementary Data S1. Methods**

**Immunohistochemistry (IHC) staining**

Three micrometer sections from formalin-fixed, paraffin-embedded aorta and TAB were deparaffinized and rehydrated, followed by antigen retrieval in Tris-EDTA buffer (pH=9) or sodium citrate buffer (pH=6). Tissues were incubated with primary antibodies against CD90, FAP, PDPN, CD248, α-SMA, PDGFRA, TGF-β, FGF21, and PDGFB (Supplementary Table S1). After endogenous peroxidase blockade was performed, tissues were subsequently incubated with secondary antibody for 40 min, DAB for visualization, and hematoxylin for counterstaining.

Four layers of the inflamed temporal arteries (adventitia, media, media-intima, and inner intima) and three layers of non-inflamed temporal arteries and aorta tissues (adventitia, media, and intima) were scored for fibroblast subtype distribution (Supplementary Figure S1). Isotype controls for IHC and negative controls for immunofluorescence are shown in Supplementary Figure S2. The percentage of positive cells per layer was assessed and calculated by Qupath 0.3.0.

Intima thickness score was calculated as the intima thickness divided by the distance from the center of the lumen to the inner border of the media. Based on the intimal thickness score, the degree of intimal hyperplasia was divided into severe occlusion (>0.7) and mild occlusion (<0.7)[1].

**Immunofluorescence (IF) staining**

IF staining was performed to study various fibroblast phenotypes and their proliferation in GCA-affected vessels. For the list of antibodies and related protocols, see Supplementary Table S2. Image cubes were captured at a magnification of 200× using the Nuance Multispectral Imaging System 3.0.1 (PerkinElmer, Waltham, MA, USA) with NuanceFX 3.0.1 software (PerkinElmer) (440:460 for DAPI=blue, 490:530 for Alexa 488=green, 570:600 for Alexa 568=red, 710:720 for Alexa 647=yellow).

**Masson’s trichrome staining**

Masson’s trichrome staining was performed to evaluate collagen deposit in blood vessels. Sections were deparaffinized in xylene and rehydrated with 100%, 96% and 70% ethanol, followed by Weigert's iron hematoxylin, Biebrich scarlet-acid fuchsin, phosphomolybdic-phosphotungstic acid and aniline blue. The proportion of collagen (blue areas) was assessed by Qupath 0.3.0. Data are presented as total collagen area per total vessel area and adventitial/media/intimal collagen areas per total vessel area.

**Real-time quantitative polymerase chain reaction (qPCR) analysis**

Total RNA was isolated from cells by TRIzol^TM^, and complementary DNA was obtained by reverse transcription using the PrimeScript RT Master Mix Kit (Invitrogen). qPCR was performed using Taqman and an ABI 7500 fast Real-Time PCR system. The relative standard curve method (2-^△△CT^) was used to determine the relative mRNA expression with probes targeting FAP (Hs00990791_m1, ThermoFisher) and IL-6 (Hs00174131_m1, ThermoFisher). Gene expression was normalized to that of GAPDH (Hs99999905_m1, ThermoFisher). In vitro experiments for qPCR analysis were repeated 3-4 times.

**Protein extraction and western blotting**

Cells were lysed in ice-cold RIPA buffer supplemented with phenylmethylsulfonyl fluoride (PMSF). Proteins were separated by sodium dodecyl sulfate-polyacrylamide gel electrophoresis (SDS-PAGE) and transferred to polyvinylidene difluoride (PVDF) membranes. Membranes were blocked in blocking buffer (LI-COR) for one hour, followed by incubation with a primary antibody cocktail (Supplementary Table S3) at 4 °C overnight, washed with TBST, and incubated with a secondary antibody cocktail (IRDye^®^ 800CW goat anti-mouse IgG, IRDye^®^ 680CW goat anti-rabbit, LI-COR) for one hour at room temperature (RT). Immunofluorescence signals were detected using an Odyssey system. The band intensities were measured using ImageJ software. The relative expression of the target proteins was normalized to that of GAPDH.

Reference.

1. van Sleen Y, Jiemy WF, Pringle S, van der Geest KSM, Abdulahad WH, Sandovici M*, et al*. A Distinct Macrophage Subset Mediating Tissue Destruction and Neovascularization in Giant Cell Arteritis: Implication of the YKL-40/Interleukin-13 Receptor alpha2 Axis. *Arthritis Rheumatol* 2021; 73(12):2327-2337.
